# Psychiatric Comorbidities Affect the Hospitalization Course of Parkinson’s Disease Patients: A Cross-Sectional Inpatient Study

**DOI:** 10.7759/cureus.16255

**Published:** 2021-07-08

**Authors:** Vaishalee Namdev, Goher Haneef, Asma T Khan, Sayeda A Basith, Anuj Virani, Johanna S Canenguez Benitez, Albulena Sejdiu, Keerthika Mathialagan, Pradipta Majumder

**Affiliations:** 1 Medicine and Surgery, Mahatma Gandhi Memorial Medical College, Indore, IND; 2 Internal Medicine, University of Health Sciences, Lahore, PAK; 3 Emergency Medicine, University of Cincinnati Medical Center, Cincinnati, USA; 4 Internal Medicine, Larkin Community Hospital, South Miami, USA; 5 Psychiatry and Behavioral Sciences, Medical University of the Americas, Charlestown, KNA; 6 Family Medicine, Windsor University School of Medicine, Cayon, KNA; 7 Internal Medicine, Larkin Community Hospital, Houston, USA; 8 Psychiatry, Saints Cyril and Methodius Hospital, Kumanovo, MKD; 9 Psychiatry, Sree Balaji Medical College and Hospital, Chennai, IND; 10 Psychiatry, Drexel University College of Medicine, Philadelphia, USA; 11 Psychiatry, WellSpan Health, York, USA

**Keywords:** parkinsons disease, affective disorders, depression, cost of hospitalization, extended hospitalization stay, suicidal behavior

## Abstract

Objectives

We aim to delineate the differences in demographic characteristics and hospitalization outcomes including the severity of illness, hospitalization length of stay (LOS) and cost, utilization of deep brain stimulation (DBS), and disposition in Parkinson’s disease (PD) inpatients with psychiatric comorbidities versus without psychiatric comorbidities.

Methods

We conducted a cross-sectional study using the Nationwide Inpatient Sample (NIS), included 56,844 PD inpatients (age ≥40 years), and subdivided them by inpatients into those without psychiatric comorbidities (N = 38,629) and with psychiatric comorbidities (N = 18,471). We compared the distributions of demographic characteristics and hospitalization outcomes (severity of illness, utilization ofDBS, and disposition) by performing Pearson’s chi-square test, and we measured the differences in continuous variables (i.e., age, LOS, and cost) by using the independent samples t-test.

Results

A significantly higher proportion of PD inpatients with psychiatric comorbidities were female (44.4%) and white (83%) and had a moderate loss of functioning (48.8%) compared to those without psychiatric comorbidities. PD inpatients with psychiatric comorbidities had an increased mean LOS (4.7 days vs. 3.7 days, P <0.001) but a lower mean cost ($37,445 vs. $ 41,957, P <0.001). Also, there was a significantly lower utilization of DBS in PD inpatients with psychiatric comorbidities (19.2% vs. 26.9%, P <0.001) compared to those without psychiatric comorbidities, and an adverse disposition of transfer to a skilled nursing facility/intermediate care facility (47.1% vs. 39.6%, P <0.001) compared to PD inpatients without psychiatric comorbidities.

Conclusion

Although PD patients with psychiatric comorbidities had a moderate loss of functioning, there was significant underutilization of DBS. Meanwhile, psychiatric comorbidities among PD patients led to increased LOS and transfer to skilled facilities.

## Introduction

Parkinson’s disease (PD) is a progressive neurodegenerative disease with a range of motor and non-motor symptoms. According to the Parkinson’s Foundation, nearly one million people live with PD in the United States (US), and almost 60,000 new cases of PD are diagnosed every year [1]. The disease appears to disproportionately affect men, who are 1.5 times more likely to have the disease than females. The incidence of PD increases with age. Even though PD is a disease of the elderly, close to 4% of PD cases are younger than 50 years of age [1]. PD is more prevalent among Whites and Hispanics, followed by Asians and Blacks [2].

Patients with PD are susceptible to various comorbid neuropsychiatric conditions. Our study explores psychiatric comorbidities in PD. Common psychiatric comorbidities found in PD are depression, anxiety, and psychosis [3]. Some of the other common psychiatric and behavioral disorders include cognitive impairment, impulse control disorders, dysthymia, sleep, and sexual disorders [4]. Additionally, with the long-term use of pharmacological treatment, delirium and psychosis are also becoming more common. Mood disorders, especially depression, often predate the development of motor symptoms in PD [3].

A systematic review demonstrated that nearly half of the patient population with PD experiences depressive symptoms [5]. Recent studies have shown that the frequency of major depression is 7-19%. Meanwhile, 10-30% of patients with PD have subtle forms of depression [5, 6]. Risk factors for depression in PD patients include female sex, a personal or family history of depression, early-onset of disease, “atypical” parkinsonism, and other psychiatric comorbidities [6]. A recent meta-analysis has found out that the point prevalence of any anxiety disorder is 31%. Generalized anxiety disorder (14.1%) is the most common form of anxiety disorder, followed by social phobia (13.8%) and anxiety disorder not otherwise specified (13.3%) [7]. There are four classes of affective symptoms in PD patients: individuals with a low probability of affective symptoms (60.4%), individuals with anxiety alone (22%), individuals with anxiety and co-existing depressive symptoms (8.6%), and prominent depressive symptoms without anxiety (9%) [8]. A recent study has shown that up to 40% of patients with PD experience generalized anxiety disorder, panic attacks, and social phobia [6]. The prevalence of apathy and emotional lability in patients with PD is 40% and 10%, respectively [6].

The pathophysiology of these neuropsychiatric symptoms is multifaceted and includes alterations in the monoaminergic system. The noradrenergic and serotonergic circuits, as well as the degeneration of dopaminergic neurons, are commonly implicated in PD patients [9]. Unlike depression, the symptoms of mania and hypomania are directly associated with deep brain stimulation (DBS) [10]. They are mediated through the anterior cingulate cortex (ACC) and its functional connectivity with the substantia nigra [11]. Anxiety in PD is often a result of a pathological process rather than a response to disability. However, in social anxiety disorder (SAD), there is a positive correlation with increased density of dopamine transporters (DAT) compared to patients without SAD, thereby suggesting the role of dopamine dysfunction in this subset of patients [12].

Psychiatric comorbidities in PD patients are often associated with faster cognitive and motor decline, increased mortality, and caregiver burnout. Depression is one of the most significant determinants in the deterioration of quality of life (QoL), followed by anxiety, apathy, aggression, and irritability [13]. Higher rates of institutionalization, faster progression to dementia, and rapid motor decline are seen in PD patients with comorbid depression compared to patients without depression. There is a higher risk of suicide in patients who have PD with psychiatric comorbidities [14]. Poor adherence to antidepressants in patients with comorbid mood disorders has also increased all-cause mortality [15]. Early recognition and treatment of psychiatric comorbidities in PD could significantly improve the QoL, reduce the morbidity and mortality associated with the disease, and alleviate the substantial financial burden on the healthcare system [13]. The goal of our study is to delineate the differences in demographic characteristics and hospitalization outcomes, including the severity of illness, length of stay (LOS) and cost, utilization of DBS, and disposition in hospitalized PD patients by psychiatric comorbidities.

## Materials and methods

Study sample

We conducted a cross-sectional retrospective study using the Nationwide Inpatient Sample (NIS). The NIS is the largest inpatient database and represents a sample of 4,411 non-federal community hospitals across 44 states in the United States [16]. In this study, we included 56,844 patients (age ≥40 years) with a primary discharge diagnosis (dx1 variable in the NIS) of PD. The sample was further subdivided into two groups: PD inpatients without psychiatric comorbidities (N = 38,629) versus with psychiatric comorbidities (N = 18,471). The International Classification of Diseases, Ninth Revision, Clinical Modification (ICD-9-CM) diagnostic codes for PD and psychiatric comorbidities are shown in Table 1.

**Table 1:** Diagnostic codes used in the study sample ICD-9-CM: International Classification of Diseases, Clinical Modification

Diagnosis	ICD-9-CM diagnostic codes
Parkinson’s disease	332.0
Mood disorders	293.83, 296.00-296.06, 296.10-296.16, 296.20-296.26, 296.30-296.36, 296.40-296.46, 296.50-296.56, 296.60-296.66, 296.7, 296.80-296.82, 296.89, 296.90, 296.99, 300.4 or 311
Anxiety disorders	293.84, 300.00-300.02, 300.09, 300.10, 300.20-300.23, 300.29, 300.3, 300.5, 300.89, 300.9, 308.0-308.4, 308.9, 309.81, 313.0, 313.1, 313.21, 313.22, 313.3, 313.82 or 313.83
Psychotic disorders	293.81, 293.82, 295.00-295.05, 295.10-295.15, 295.20-295.25, 295.30-295.35, 295.40-295.45, 295.50-295.55, 295.60-295.65, 295.70-295.75, 295.80-295.85, 295.90-295.95, 297.0-297.3, 297.8-298.4, 298.8 or 298.9
Substance use disorders	292.0, 292.82-292.89, 292.9, 304.00-304.93, 305.20-305.93 or 648.30-648.34

Variables

Psychiatric comorbidities were defined by various co-diagnoses in the patient’s records. We included mood disorders, anxiety disorders, psychotic disorders, and substance use disorders (SUD) as the entities of interest [17]. We calculated the LOS as the number of nights the patient was hospitalized for the management of primary discharge diagnosis (dx1) of PD. We also included the total cost during hospitalization, excluding professional fees and non-covered charges. The hospitalization outcomes of interest included in this study are the severity of illness, which measures the loss of body functions (minor, moderate, or major) using the All-Patient Refined Diagnosis-Related Groups (DRGs) software developed by Health Information Systems (3M™ APR DRG Software, Health Information Systems, Salt Lake City, UT), and utilization of DBS (identified using ICD-9-CM procedure code 02.93), disposition status (routine, transfer to short-term hospital, transfer to skilled nursing facility/intermediate care facility (SNF/ICF), home health care, against medical advice and died) [18].

Statistical analysis

We compared the distributions of demographic characteristics and hospitalization outcomes (severity of illness, utilization of DBS, and disposition) in PD inpatients without versus with psychiatric comorbidities by performing descriptive statistics and Pearson’s chi-square test. Next, we measured the differences in continuous variables (i.e., age, LOS, and cost) between PD inpatients without versus with psychiatric comorbidities using the independent sample t-test. All analyses were conducted using the Statistical Package for the Social Sciences (SPSS) version 26.0 (IBM Corp., Armonk, NY) and statistical significance was set at a two-sided P value of <0.05.

Ethical approval

The NIS is publicly available de-identified data that protects patients, physicians, and hospital-related information; hence, we were not required to take institutional review board permission for our study [16].

## Results

**Figure 1:**
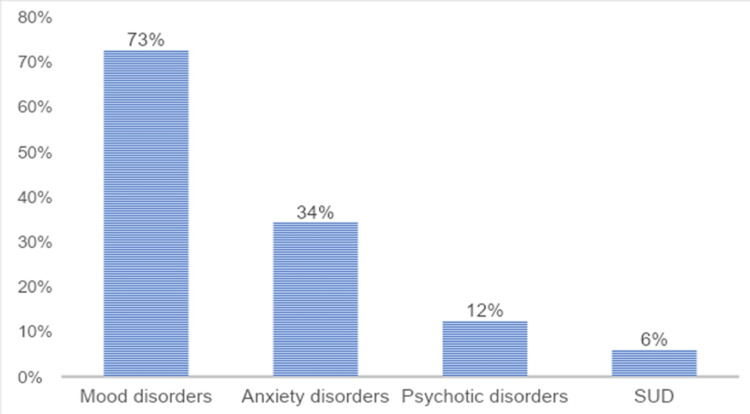
Distribution of psychiatric comorbidities in Parkinson’s disease inpatients SUD: substance use disorder

Our study included 56,844 PD inpatients, and 18,471 inpatients (32.5%) had psychiatric comorbidities. Among psychiatric comorbidities, a higher proportion of PD inpatients had mood disorders (72.7%) and anxiety disorders (34.3%) followed by psychotic disorders (12.4%) and SUD (6%) as shown in Figure 1.

The mean age of PD inpatients with psychiatric comorbidities was 70.7 years. A significantly higher proportion of PD inpatients with psychiatric comorbidities were female (44.4%) and white (83%) compared to PD inpatients without psychiatric comorbidities. There was statistically no significant difference between the groups based on the median household income (P = 0.150).

A higher proportion of PD inpatients with psychiatric comorbidities had a moderate loss of functioning (48.8% vs. 42.2%, P <0.001), and the mortality rate was lower (0.8% vs. 1.4%, P <0.001) compared to PD inpatients without psychiatric comorbidities. PD inpatients with psychiatric comorbidities had an increased mean LOS (4.7 days vs. 3.7 days, P <0.001) but a lower mean cost ($37445 vs. $ 41957, P <0.001). There was a significantly lower utilization of DBS in PD inpatients with psychiatric comorbidities (19.2% vs. 26.9%, P <0.001) compared to those without psychiatric comorbidities.

A significantly higher proportion of PD inpatients with psychiatric comorbidities were transferred to SNF/ICF (47.1% vs. 39.6% in PD inpatients without psychiatric comorbidities), as shown in Table 2.

**Table 2:** Differences in Parkinson’s disease inpatients by psychiatric comorbidities SD: standard deviation; DBS: deep brain stimulation; SNF: skilled nursing facility; ICF: intermediate care facility

Variable	Psychiatric comorbidities		P value
	no	yes	
Number of inpatients	38629	18471	-
Mean age (SD), in years	72.8 (10.9)	70.7 (10.8)	<0.001
Sex, in %			
Male	66.4	55.6	<0.001
Female	33.6	44.4	
Race, in %			
White	79.4	83.0	<0.001
Black	7.5	4.7	
Hispanic	6.6	7.1	
Others	6.5	5.2	
Median household income, in %			
0 – 25^th^ percentile	22.4	23.3	0.150
26^th^ – 50^th^ percentile	25.1	24.3	
51^st^ – 75^th^ percentile	25.3	25.4	
76^th^ – 100^th^ percentile	27.2	27.0	
Severity of illness, in %			
Minor loss of function	35.6	29.4	<0.001
Moderate loss of function	42.2	48.8	
Major loss of function	22.2	21.8	
Hospitalization outcomes			
Mean length of stay (SD), in days	3.7 (6.3)	4.7 (9.8)	<0.001
Mean cost (SD), in $	41957 (46161)	37445 (38597)	<0.001
Utilization of DBS, in %	26.9	19.2	<0.001
Disposition			
Routine	43.7	36.1	<0.001
Transfer to SNF/ICF	40.6	48.6	
Home health care	13.8	14.1	
Against medical advice	0.4	0.3	
Died	1.4	0.8	

## Discussion

In our study, 32.5% of the patients with PD had psychiatric comorbidity, of which 72.7% had symptoms of mood disorders, followed by anxiety disorders (34.3%). Symptoms of depression and anxiety could be early prodromal manifestations of PD [19]. Risk factors for depression in PD include female sex, personal and familial history of depression, early-onset PD, "atypical" parkinsonism, and other psychiatric comorbidities [6]. One of the biggest hurdles in diagnosing comorbid depression in PD is the overlap between the psychosomatic symptoms of depression and PD. The national institute of health recommends an inclusive approach, where the somatic symptoms are considered a part of depression [20]. Anxiety is often comorbid with depression in patients with PD. The most common manifestation in patients with PD is a generalized anxiety disorder, followed closely by panic disorder and social phobias, which reduce QoL [6, 21]. PD is also associated with situational phobias related to motor symptoms, for instance, freezing of gait and tremors in social situations [21]. 

Subsequently, we also found a higher prevalence of psychotic disorders and SUD among PD patients in our study. Some of the risk factors for psychosis in PD patients are advanced age, longer disease duration, advanced disease, disorders of the sleep-wake cycle, excessive dopamine therapy, a high degree of cognitive impairment, and a family history of dementia [22, 23]. Before dopamine replacement therapy (DRT), the prevalence of psychotic symptoms in untreated PD was less than 10% [24]. Psychosis is a crucial determinant of hospitalization and nursing home placement in patients with PD, which plays a significant part in increased caregiver burnout. Repeated hospitalization also leads to a disruption in the patient's medication schedule leading to accelerated cognitive decline [6]. Visual hallucinations are the most reported symptoms, followed by auditory, tactile, and even olfactory hallucinations [22, 23].

Substance-related addictions, commonly known as dopamine disorder syndrome (DDS), are common among PD patients who use higher doses of dopamine than required. Pathology of the dopaminergic circuit is common to both PD and substance use disorders. DDS is a neuropsychiatric behavioral syndrome, where a disturbance in the impulse control system leads to addictive behaviors. Current estimates show that substance-related addiction in PD is due to DDS, and the most common substances of abuse are apomorphine and levodopa [25].

In our study, PD patients with psychiatric comorbidities had a moderate loss of functioning. Our results are consistent with a previous study, which reported pronounced cognitive decline and deterioration in activities of daily living [26]. Although PD patients with psychiatric comorbidities had a moderate loss of functioning, our study found a significant underutilization of DBS. DBS has shown promising results in treating motor fluctuations in moderate to severe PD. Several factors could be implicated in the underutilization of DBS by patients like misinformation about indications, side effects and cost, and insurance coverage burden [27].

Our study showed increased mean LOS in patients with psychiatric comorbidities and higher transfer rates to SNF/ICF. Depression in PD is often associated with worse QoL, increased cognitive impairment, and caregiver burden, leading to increased transfer to skilled facilities and an increased mean LOS [28]. Our study also found a lower mortality rate among PD inpatients with psychiatric comorbidities. Results from past studies on the effect of neuropsychiatric symptoms on mortality rates among PD patients have been conflicting. In addition to complications arising from PD, the progressive and degenerative nature of the disease by itself leads to increased mortality rates [29].

As a cross-sectional study, one of our limitations was that we could not find a causal relationship between the development of psychiatric comorbidities and the primary pathophysiology of PD. In addition to this, another limitation was that we used an administrative database and therefore lacked patient-level clinical information, as the patients were identified based on diagnostic codes rather than symptoms themselves, which can lead to underdiagnosis of the psychiatric comorbidities. We used the NIS, which enabled us to present data of a larger patient population, increasing the power of our study with minimal reporting bias.

## Conclusions

PD patients with psychiatric comorbidities had a moderate loss of functioning, yet, there was significant underutilization of DBS. Psychiatric comorbidities led to extended hospitalization stays and higher disposition rates to nursing facilities. Further studies to assess the impact of psychiatric comorbidities in PD are essential for earlier diagnosis and management and to improve the QoL and cognitive functioning in this at-risk population.
